# Use of live *Variola virus* to determine whether CAST/EiJ mice are a suitable surrogate animal model for human smallpox

**DOI:** 10.1016/j.virusres.2019.197772

**Published:** 2019-10-05

**Authors:** Nadia F. Gallardo-Romero, Christina L. Hutson, Darin Carroll, Ashley V. Kondas, Johanna S. Salzer, Sharon Dietz-Ostergaard, Scott Smith, Paul Hudson, Victoria Olson, Inger Damon

**Affiliations:** a Centers for Disease Control and Prevention, National Center for Emerging and Zoonotic Infectious Diseases, Division of High-Consequence Pathogens and Pathology, Poxvirus and Rabies Branch, 1600 Clifton Rd. NE, Atlanta, GA, 30333, USA; b Centers for Disease Control and Prevention, National Center for Emerging and Zoonotic Infectious Diseases, Division of Scientific resources, Comparative Medicine Branch, 1600 Clifton Rd. NE, Atlanta, GA, 30333, USA

**Keywords:** Orthopoxvirus, Smallpox, Variola virus, CAST/EiJ mice, Immune response

## Abstract

Numerous animal models of systemic orthopoxvirus disease have been developed to evaluate therapeutics against variola virus (VARV), the causative agent of smallpox. These animal models do not resemble the disease presentation in human smallpox and most used surrogate *Orthopoxviruses*. A rodent model using VARV has a multitude of advantages, and previous investigations identified the CAST/EiJ mouse as highly susceptible to monkeypox virus infection, making it of interest to determine if these rodents are also susceptible to VARV infection. In this study, we inoculated CAST/EiJ mice with a range of VARV doses (10^2^-10^6^ plaque forming units). Some animals had detectable viable VARV from the oropharynx between days 3 and 12 post inoculation. Despite evidence of disease, the CAST/EiJ mouse does not provide a model for clinical smallpox due to mild signs of morbidity and limited skin lesions. However, in contrast to previous rodent models using VARV challenge (i.e. prairie dogs and SCID mice), a robust immune response was observed in the CAST/EiJ mice (measured by Immunoglobulin G enzyme-linked immunosorbent assay). This is an advantage of this model for the study of VARV and presents a unique potential for the study of the immunomodulatory pathways following VARV infection.

## 1. Introduction

Smallpox is the only human disease that has been successfully eradicated through massive vaccination campaigns. An intensified global effort, led by the World Health Organization (WHO), allowed for this devastating disease to be declared eradicated in 1980. The campaign used live vaccinia virus, which like the causative agent of smallpox, variola virus (VARV) offers a cross protection against all members of the *Orthopoxvirus* genus. Almost 4 decades has passed since the declaration of smallpox eradication, and routine vaccinations have ceased, leaving an increasing percentage of the current human population worldwide susceptible to an *Orthopoxvirus* infection.

Following smallpox eradication, all materials that were identified as potentially containing VARV, as well as declared VARV stocks, were transferred to one of two WHO approved repositories: The State Centre for Research on Virology and Biotechnology (VECTOR) in Novosibirsk, Russia, and the Centers for Disease Control and Prevention (CDC) in Atlanta, Georgia, United States of America. From that time, the World Health Assembly (WHA) has discussed the timing of destruction of VARV. Concern persists over the potential use of VARV as a biological threat (Fleck, 2003). Therefore, considerable research efforts have focused on the generation of safer and effective vaccines as well as the evaluation of potential antiviral compounds for their efficacy against VARV. Although initial assessment is conducted *in vitro*, determination of medical countermeasure effectiveness against smallpox disease optimally would be characterized against the authentic agent within an established animal model of smallpox disease.

In 1999 the Institute of Medicine (IOM) formed a committee that supported the public health need to develop medical countermeasures against smallpox. In 2009, the committee on Assessment of Future Scientific Needs for Live Variola Virus hosted by the IOM reviewed all research conducted between 1999 and that date, and concluded that the development of medical countermeasures against smallpox remained of grand importance due the pandemic potential should VARV be released due to deliberate or accidental actions. The scientific uses of live VARV were evaluated in four different areas, including development of therapeutics, development of vaccines, genomic analysis, and discovery research. The committee concluded that the development of animal models would be of great use to assess the efficacy of antivirals and third-generation vaccines (Medicine, 2009). Studies have shown disease within the nonhuman primate model; however, in order to induce illness in nonhuman primates, the required infectious dose (1 × 10^8^-1 × 10^9^ VARV virions) is required to be given intravenously and is much greater than the dose associated with a natural infection. This high dose and unnatural route of inoculation bypasses the early respiratory tract/lymphatic tissue replication, the first viremia, and the long incubation period (~12–17 days) before presentation of early clinical signs of infection in humans (Jahrling et al., 2004; Wahl-Jensen et al., 2011; Mucker et al., 2013). New animal models that could more closely mimic key aspects of the human disease incubation and progression will help to better characterize and develop utilization strategies for therapeutics and vaccines for use as countermeasures against smallpox.

Other surrogate models for smallpox have been well studied but all of them lack of either skin lesion presentation or the disease progression is very different from human smallpox. The prairie dog-monkeypox model presents a prolonged incubation period and disseminated skin lesions, having the potential for study of virulence factors, therapeutics, and vaccine efficacy (Hutson et al., 2009), however there are limitations to this model. Prairie dogs cannot be infected with VARV, therefore precluding the ability to evaluate the authentic agent of smallpox, and are not inbred laboratory raised animals, which present individual host variabilities and lack of reagents for extensive molecular and serological evaluation (Carroll et al., 2013).

In general, analogous to VARV studies in adult mice, inbred immunocompetent mouse strains are relatively difficult to infect with Monkeypox virus (MPXV) and observe symptomatic illness. A survey of a large panel of inbred mouse strains, wild-derived, identified the CAST/EiJ mouse as highly susceptible to infection with MPXV (Americo et al., 2010). Unpublished data from the same laboratory has suggested that CAST/EiJ mice are highly susceptible to a wide range of *Orthopoxviruses* at lower infectious doses than seen in other inbred mouse strains. Previous studies demonstrated that intranasal infection of CAST/EiJ mice with MPXV resulted in successful viral replication in internal organs, however, a deficiency in the production of interferon gamma in lung, was identified and concluded that it was a characteristic feature of the high sensitivity of CAST/EiJ mice to MPXV infection (Earl et al., 2012). The utility of a novel inbred mice strain, with minimal intrinsic variability, and greater availability of specific immunologic reagents, that is easy to handle and maintain, and present similar clinical signs than human smallpox, will improve our ability to evaluate medical countermeasures using a more appropriate model than what currently exist.

## 2. Materials and methods

All work with live VARV was conducted within the maximum containment biosafety level 4 (ABSL4) laboratory, under the Terms of Reference of the WHO CC for Smallpox and Other Poxvirus Infections at the WHO CC at the CDC in Atlanta, GA USA. The facility is regularly reviewed for biosafety and biosecurity practices by independent U.S. and WHO teams.

### 2.1. Animals

CAST/EiJ female mice were obtained from the Jackson laboratory (Bar Harbor, ME. Stock number 000928). Animals were group housed in a ventilated cage system, with aerosol filter tops. Standard mouse husbandry practices were performed during the experiment in accordance with CDC Institutional Animal Care and Use Committee (IACUC) guidelines under the approved protocol 2379DAMMOUC. In addition to mouse chow, all animals received oats as appetence monitor, as well as a plastic nest and enrichment nesting materials.

For the first phase of this experiment, thirty mice were received at 7–8 weeks of age and divided in groups of five mice per viral dose [10^2^-10^6^ plaque forming units (pfu)], three control mice received the equivalent to 5 × 10^5^ pfu of inactivated VARV (gamma irradiated 4.4 × 10^6^ rads), and two control mice received diluent only (PBS + 0.05% BSA) (Table 1). During this phase, the investigators were aware of the inoculation dose the animals received.

For the second phase, twenty-six mice were received at 4–5 weeks old. The lower viral dose (10^2^ pfu) group was excluded and the rest of the groups were similar to the first phase, apart from the addition of one mouse in the diluent group. During this phase, the investigators were blinded regarding the viral dose used for inoculation of each group of animals.

After 72 h of acclimation in a biosafety level 2 facility, a blood sample from the submandibular vein and an oral swab were taken to ensure the absence of *Orthopoxvirus* antibodies and DNA. Then animals were then moved to the ABSL4 laboratory. The animals were acclimated for an additional 72 h before viral inoculation.

### 2.2. Virus and inoculum preparation

The Harper strain of *Variola virus* (VARV JAP51_hrpr) was used for this study, as it has been established for use in non-human primate studies (Jahrling et al., 2004b; Mucker et al., 2013; Wahl-Jensen et al., 2011b). Crude virus was semi-purified by 2–3 1, 1, 2 tri chlorofluoroethane extractions followed by ultracentrifugation through two sucrose cushion (Esposito et al., 1978). To mimic natural infection to the greatest extent feasible, VARV was administered by the intranasal route. The inoculum was diluted in 10 μl (5 μl per nostril) of phosphate-buffered saline (PBS) + 0.05% bovine serum albumin (BSA) to achieve 5 different viral doses (5 × 10^2^, 5 × 10^3^, 5 × 10^4^, 5 × 10^5^ or 5 × 10^6^ pfu). Mice were anesthetized with 1–5% isoflurane gas during inoculation on day zero. All the following days were recorded as day 1–21 post inoculation (pi).

### 2.3. Specimen collection and preparation

All animal handling and sampling was done while mice were anesthetized with 1–5% isoflurane gas. The mice were initially induced in their cage using 5% isoflurane and then transported to the down draft table where they were maintained in a surgical plane of anesthesia on a nasal cone/face mask for sampling and data collection. Oral, anal, ocular, and lesion swabs (if present), were systematically collected during the first phase study on days 3, 5, 7, 10, 12, 14, 17, and 19. In the second phase, only oral samples were routinely collected on days 2, 4, 7, 9, 11, 14, 16, and 18. Oral swabs were taken with three rotations per cheek, one rotation over the palate, and another rotation over the tongue. Ocular swabs were passed over each closed eye three times, and the anal region was swabbed three times. Ocular and anal swabs were not collected during the second phase of the study. Every sample day, the animals were weighed and underwent a thorough skin inspection to identify potential lesions.

Daily observations of each animal’s food consumption, activity level, and general appearance were recorded. General appearance of animals were observed before handling and/or after recovery from anesthesia. Euthanasia was performed under anesthesia with 5% isoflurane gas by intracardiac exsanguination followed by cervical dislocation on day 21 pi, compliant with the CDC IACUC approved protocol.

Necropsies were performed on each animal and samples (brain, lung, liver, spleen, ovaries, heart, and kidney) were homogenized using the GenoGrinder 2000 (SPEX Sample Prep). Only spleen and ovaries where tested in the second phase of the study, apart from two animals that died or were euthanized during the study, from these animals all organs mentioned before were tested. Swabs were processed using the Swab Extraction Tube System (Part # 03315568001, Roche Diagnostics, Basel Switzerland). The samples were inactivated in the ABSL4 lab using approved inactivation procedures. In brief, the homogenates/swab eluates were placed in lysis buffer and fully submerged in a 56 °C water bath for 15 min prior to removal from the ABSL4 laboratory and transfer to the BSL2 laboratory for processing. The extraction of viral DNA was performed using Qiagen tissue kits on the BioRobot® EZ1 workstation, according to the manufacturer’s instructions. Serum was separated from blood and inactivated with gamma irradiation (4.4 × 10^6^ rads) prior to further testing in the BSL2 laboratory.

### 2.4. Viral DNA analysis

All samples were tested in duplicate using the A36R real time PCR assay (Kondas et al., 2015a, b), which targets an envelope protein gene. In addition to the sample, every reaction plate contained both a positive and negative control; the positive control consisted of a standard curve of serial 10-fold dilutions of VARV DNA (from 500 pg to 5 fg) and the negative control consisted of deionized, demineralized water. A sample with CT value (the cycle when fluorescence crossed the threshold) of < 40 was considered positive to contain viral DNA in the sample, and further tested for viral viability.

### 2.5. Virus-tissue infectivity

BSC-40 cell monolayers were inoculated with 10-fold dilutions of sonicated tissue homogenate or swab eluate. Infected cells were incubated at 35.5 °C in a 6% CO_2_ atmosphere in semi-solid medium (Roswell Park Memorial Institute medium + 1% carboxymethylcellulose, 2% fetal bovine serum, and 1% penicillin/streptomycin). At 96 h pi, cells were stained with 2X crystal violet and plaques were counted to determine the viral titer in plaque forming units/mL (pfu/mL). A sample was considered positive (containing viable virus) if the plaques average of the duplicates was ≥ 5.

### 2.6. Serological analysis

The enzyme-linked immunosorbent assay (ELISA) was used for detection of VARV immunoglobulin type G (IgG). The VARV Bangladesh strain 7124 (gamma irradiated 1.32 × 10^7^ rads) was utilized to coat the microtiter plates, at a concentration of 0.3 μg/ml diluted in carbonated buffer 7.4 pH. Plates were incubated overnight at 4 °C. Animal sera were tested at an initial dilution of 1:50 with fourfold dilutions to 1:3200. One hundred μl per well of a 1:2000 dilution of goat anti-mouse IgG (Part #1721011, Bio-Rad Laboratories. California, USA) was used as conjugate. Negative murine ascitic fluid was used as negative control, and sera from California mice previously infected with *Volepox virus*, were used as positive controls (Gallardo-Romero et al., 2012). The average of all optical densities values from the negative controls, plus two standard deviations, was used to generate a cut-off value (COV). A sample was considered positive if the average of the duplicates had values over the cut off in at least two consecutives dilutions (1:50 and 1:200).

## 3. Results

### 3.1. Phase 1 (mice inoculated at 8–9 weeks old)

During the first four days after inoculation, no clinical signs of disease were noted. Weight loss was not a major component of clinical illness. A 15% weight loss was observed in 1/3 animals challenged with gamma-inactivated virus on day 7 pi, 5% weight loss was documented in 1/5 and 2/5 animals challenged with 10^5^ and 10^6^ pfu VARV, respectively on day 9 pi (data not shown). Other clinical signs (decreased activity, reduced grooming, erythema, ocular discharge, and skin lesions) are summarized in Table 1.

Evidence of virus shedding in oral, ocular, and anal swabs eluates was evaluated. Based on the PCR results from the swabs collected from the oral cavity, viral DNA was detected in oral swabs in a dose dependent manner, with all mice in the highest virus challenge group displaying at least one oral swab with VARV DNA (Table 2). Viable virus was identified in a subset of animals challenged with 10^6^ pfu between 3 and 7 days post challenge, where 3 of 5 animals shed viable VARV in oral and ocular secretions on days 3 and 7 pi (Table 2), consistent with clinical signs presentation (Table 1). Additionally, one animal from the lowest dose challenge group (10^2^ pfu) shed viable VARV in oral cavity on day 12 pi.

Low levels of viral DNA were detected from the oropharynx, ocular, and anal secretions from animals challenged at 8–9 weeks of age. Viral DNA was detected between days 3–14 pi, and infectious virus was < 1 × 10^3^ pfu/mL.

All the animals survived the infection and were euthanized on day 21 pi. Ovaries and spleen were tested from all animals and only one animal (#21 from group challenged with 10^4^ pfu) had viral DNA in ovarian tissues at day 21 pi (Ct 34); although, viable virus was not detected in the tissue. No other ovaries and spleens showed evidence of detectable virus by either real time PCR or tissue culture.

All animals exposed to 10^3^ or higher pfu of live VARV showed a strong immune response by day 21 pi. Two out of five animals exposed to 10^2^ pfu showed a minimum antibody response while the other three animals in the group produced a robust IgG response, similar to higher pfu groups. Only one of the animals exposed to inactivated VARV showed a very limited immune response. The serological results of the first phase of this study are presented in Table 3.

### 3.2. Phase 2 (mice inoculated at 5–6 weeks old)

For the first six days after inoculation, no clinical signs of disease were observed. Weight loss was rarely noted, and decreased activity was not observed. Other clinical signs (reduced grooming and pruritus, erythema, respiratory distress under anesthesia, ocular discharge, and skin lesions) are summarized in Table 4.

One mouse (#19 from group 10^3^ pfu) was found dead on day 15 pi without showing previous clinical signs of disease, and all the organs tested negative for VARV by real time PCR. Only one mouse (#13 from group 10^5^ pfu) reached the clinical score for euthanasia on day 16 pi due to the 20% weight loss, piloerection, respiratory distress and conjunctivitis. All the internal organs from this animal were tested, and only lung amplified viral VARV DNA by real time PCR, but was negative for viable virus (Table 5). All other animals from groups 10^4^-10^6^ pfu resolved clinical signs by day 19 pi. At day 21 pi all the animals that survived the challenge were humanely euthanized for necropsy.

Viral dissemination (including possible skin lesions), and shedding in oral secretions was observed in the animals challenged at 5–6 weeks of age more frequently than in the older animals challenged in phase 1 of the study but at similar levels of viable virus (< 1000 pfu/mL) (Table 5). Animals challenged in with 10^5^ or 10^6^ pfu shed infectious virus in their oropharynx between days 4 and 11 post infection. Infectious virus was not isolated after day 11, but viral DNA could be detected in the oropharynx through day 14 in some animals. Ocular and anal swabs were not tested in the second phase of the study. Tail lesions swabs were collected from two animals from the 10^5^ pfu group on days 11 and 14 pi. The samples yield DNA positive for VARV by real time PCR, however, viable virus at days 11 and 14 in two animals challenged with 10^5^ pfu was not isolated.

All animals challenged with live virus seroconverted with high levels of VARV IgG antibody at day 21 (> 1:3200) and similarly than phase one of the study, only one animal from the inactivated virus group showed minimal immune response. These results are shown on Table 6.

## 4. Discussion

Because smallpox was declared eradicated in 1980 and vaccination was discontinued, a large percentage of the current global population is susceptible to smallpox and other *Orthopoxvirus* infections. As VARV is still considered a potential threat due to concerns over bioterrorism (Fleck, 2003), the identification of new antiviral treatments, and safer vaccines is considered a priority for national preparedness. To truly understand the efficacy of these potential medical countermeasures, the establishment of an animal model of smallpox disease is needed. The only current model available for VARV is non-human primates infected with very high loads of VARV intravenously, which leads to a rapidly fulminant disease with the majority of animals dying during the first week post inoculation (Jahrling et al., 2004a; Mucker et al., 2013; Wahl-Jensen et al., 2011a). In contrast, human infection involves an incubation period of about a week or longer, followed by a prodrome consisting of general malaise and fever before skin rash presentation, inconsistent with many features of the non-human primate VARV model.

In this study we wanted to evaluate if CAST/EiJ mice could serve as a suitable model to study VARV infection and whether the presentation of the subjective clinical signs (including reduced grooming, weight loss, decreased activity, ocular and nasal swelling, and erythema) were dose dependent. Mice challenged intranasally with 10^5^ or 10^6^ pfu VARV manifest mild clinical illness, which led to potential systemic spread and sporadic shedding of virus. Results of viral spread to the respiratory tract were similar to another publication with VARV using ICR and SCID mice (Titova et al., 2015). In this study, the differences in clinical illness between younger and older mice was not appreciably different, although a greater number of younger animals had viable virus in their oral secretions compared to the older animals. The CAST/EiJ model will present several challenges for antiviral or therapeutic evaluation due to the minimal levels of morbidity and lack of mortality attributable to VARV infection. An ideal model would present with notable skin rash and more severe clinical signs of disease and mortality that could be compared with the treated groups for increase of survivorship and decrease of skin rash and general morbidity.

Despite evidence of disease, the CAST/EiJ mouse does not provide a model for clinical smallpox due to mild signs of morbidity and limited skin lesions. However, our investigation was able to detect a robust immune response as measured by IgG ELISA using the CAST/EiJ- VARV model. This is a clear advantage of this model for the study of VARV in contrast to previous rodent models using VARV challenge (i.e. prairie dogs and SCID mice) and presents a unique potential for the comparative study for understanding the immune response of the immunomodulatory pathways following VARV infection.

## Figures and Tables

**Table 1:** Comparison of clinical signs presentation during Phase 1 of the study, on CAST/EiJ mice infected with different doses of *Variola virus* (animals challenged at 8–9 weeks of age).

Dosage	5 × 10^2^*	5 × 10^3^*	5 × 10^4^*	5 × 10^5^*	5 × 10^6^*
Decreased activity onset (number of animals)	Day 9 pi (2/5)	Day 7 pi (4/5)	Day 7 pi (5/5)	Day 5 pi (5/5)	Day 5 pi (4/5)
Reduce grooming onset (number of animals)	Day 7 pi (5/5)	Day 7 pi (4/5)	Day 7 pi (5/5)	Day 7 pi (5/5)	Day 7 pi (5/5)
Oral/nasal erythema (number of animals)	NO	NO	Day 7 pi (2/5)	Day 7 pi (2/5)	Day 7 pi (5/5)
Ocular discharge (number of animals)	NO	Day 10 pi (1/5)	Day 7 pi (2/5)	Day 7 pi (3/5)	Day 7 pi (3/5)
Rear paw/tail skin lesions (number of animals)	NO	Day 10 pi (2/5) NP	Day 12 pi (2/5) NP	Day 12 pi (2/5)	Day 12 pi (3/5) NP
Complete recovery**	Day 14 pi	Day 17 pi	Day 17 pi	Day 19 pi	Day 19 pi

Mice from gamma irradiated VARV and vehicle (PBS + .05% BSA) groups did not show any clinical signs mentioned above.

pi = days post inoculation.

NO = Never observed.

NP = Negative by PCR.

* Challenged groups, in plaque forming units (pfu).

** Pain score back to 0 points.

**Table 2:** Evidence of viable virus (pfu/mL) and viral DNA (CT value by real time PCR) prescence in CAST/EiJ mice samples from Phase 1 (animals challenged with *Variola virus* at 8–9 weeks of age).

ID#	Group*	Day pi^˟^	Sample	PCR^ˠ^	pfu/mL
32	5×l0^2^	12	oral swab	36.7/36.4	100
32	5×l0^2^	14	oral swab	35.6/35.7	BLD
14	5×10^3^	12	oral swab	35.6/35.2	BLD
14	5×10^3^	12	ocular swab	37.5/37.6	BLD
14	5×10^3^	14	anal swab	37.3/37.8	BLD
15	5×10^3^	10	anal swab	37.2/37.1	BLD
16	5×10^3^	10	ocular swab	36.2/36.5	BLD
16	5×10^3^	14	anal swab	36.9/37.3	BLD
21	5×l0^4^	5	oral swab	35.6/34.7	BLD
21	5×10^4^	7	oral swab	38.2/38.1	BLD
21	5×10^4^	7	ocular swab	36.7/37.7	BLD
21	5×l0^4^	7	anal swab	36.7/38.4	BLD
21	5×l0^4^	10	oral swab	38.2/38.2	BLD
21	5×10^4^	10	anal swab	37.7/37	BLD
21	5×l0^4^	12	oral swab	36.4/35.5	BLD
21	5×10^4^	21	ovaries	34.0/34.1	BLD
22	5×l0^4^	10	ocular swab	39/38.9	BLD
22	5×10^4^	10	anal swab	38/38.7	BLD
5	5×10^5^	5	oral swab	38.1/38.6	BLD
6	5×10^5^	10	ocular swab	32.1/39.5	BLD
6	5×10^5^	12	oral swab	35.4/36.5	BLD
6	5×10^5^	12	anal swab	39.2/38	BLD
6	5×10^5^	12	rear paw lesion swab	38.1/38.0	BLD
6	5×10^5^	14	oral swab	37.4/36.7	BLD
6	5×10^5^	14	ocular swab	38.1/37.3	BLD
7	5×10^5^	3	ocular swab	38.3/38.7	BLD
7	5×10^5^	7	oral swab	36/36	BLD
7	5×10^5^	10	oral swab	38.1/39.2	BLD
7	5×10^5^	10	anal swab	37.9/37.2	BLD
7	5×10^5^	12	oral swab	33.4/34.2	BLD
7	5×10^5^	12	ocular swab	37.4/36.4	BLD
7	5×10^5^	12	rear paw lesion swab	38/37	BLD
8	5×10^5^	10	anal swab	37.9/38.7	BLD
1	5×l0^6^	7	oral swab	38.1/37.9	BLD
1	5×l0^6^	7	ocular swab	37/37.2	BLD
1	5×l0^6^	10	ocular swab	38.6/38.7	BLD
2	5×l0^6^	5	oral swab	38.1/38.1	BLD
2	5×l0^6^	7	oral swab	35.8/35.5	100
2	5×l0^6^	7	ocular swab	37.9/34.6	BLD
2	5×l0^6^	7	anal swab	38.7/38.9	BLD
2	5×l0^6^	10	oral swab	37.9/37.8	BLD
3	5×l0^6^	7	oral swab	37.1/38.4	BLD
3	5×l0^6^	7	anal swab	36.4/39.9	BLD
19	5×l0^6^	3	oral swab	32.5/32.6	1000
19	5×l0^6^	7	oral swab	33.2/33.3	100
19	5×l0^6^	7	ocular swab	35.2/34.7	100
19	5×l0^6^	10	ocular swab	38.9/38.4	BLD
19	5×l0^6^	10	anal swab	39.3/39.6	BLD
19	5×l0^6^	12	oral swab	37.2/37.2	BLD
20	5×l0^6^	3	oral swab	38.5/36.9	BLD
20	5×l0^6^	5	oral swab	34.2/33.9	BLD
20	5×l0^6^	7	oral swab	37.0/35.9	100
20	5×l0^6^	7	ocular swab	35.0/35.2	BLD
20	5×l0^6^	7	anal swab	38.7/37.8	BLD
20	5×l0^6^	10	ocular swab	37.9/39.1	BLD
20	5×l0^6^	10	anal swab	39.1/37.5	BLD

* Challenged groups, in plaque forming units (pfu).

˟ pi = days post inoculation, day of sample collection.

ˠ Duplicates of the positive values, number of cycles needed to cross the threshold (CT).

" Viable virus content per mL, in pfu. BLD = below limit of detection.

Samples containing viable virus are shaded in grey.

Samples from gamma irradiated VARV and vehicle (PBS + .05%BSA) groups, did not show evidence of detectable virus either by real time PCR or Tissue Culture.

**Table 3:** Immune response of 8–9 weeks old CAST/EiJ mice during Phase 1, after challenge with different doses of *Variola virus*.

ID #	Group*	Age	Serum^˟^	Positive Dilution^ˠ^
17	Diluent	9 weeks old	21 days pi	BLD
18	Diluent	9 weeks old	21 days pi	BLD
28	GAMIR	8 weeks old	21 days pi	BLD
29	GAMIR	8 weeks old	21 days pi	BLD
30	GAMIR	8 weeks old	21 days pi	1:50
31	5 × 10^2^	8 weeks old	21 days pi	> 1:3200
32	5 × 10^2^	8 weeks old	21 days pi	> 1:3200
11	5 × 10^2^	9 weeks old	21 days pi	1:50
12	5 × 10^2^	9 weeks old	21 days pi	> 1:3200
13	5 × 10^2^	9 weeks old	21 days pi	1:50
26	5 × 10^3^	8 weeks old	21 days pi	> 1:3200
27	5 × 10^3^	8 weeks old	21 days pi	> 1:3200
14	5 × 10^3^	9 weeks old	21 days pi	> 1:3200
15	5 × 10^3^	9 weeks old	21 days pi	> 1:3200
16	5 × 10^3^	9 weeks old	21 days pi	> 1:3200
21	5 × 10^4^	8 weeks old	21 days pi	> 1:3200
22	5 × 10^4^	8 weeks old	21 days pi	> 1:3200
23	5 × 10^4^	9 weeks old	21 days pi	> 1:3200
24	5 × 10^4^	9 weeks old	21 days pi	> 1:3200
25	5 × 10^4^	9 weeks old	21 days pi	> 1:3200
4	5 × 10^5^	9 weeks old	21 days pi	> 1:3200
5	5 × 10^5^	9 weeks old	21 days pi	> 1:3200
6	5 × 10^5^	8 weeks old	21 days pi	> 1:3200
7	5 × 10^5^	8 weeks old	21 days pi	> 1:3200
8	5 × 10^5^	8 weeks old	21 days pi	> 1:3200
1	5 × 10^6^	9 weeks old	21 days pi	> 1:3200
2	5 × 10^6^	9 weeks old	21 days pi	> 1:3200
3	5 × 10^6^	9 weeks old	21 days pi	> 1:3200
19	5 × 10^6^	8 weeks old	21 days pi	> 1:3200
20	5 × 10^6^	8 weeks old	21 days pi	> 1:3200

BLD = below limit of detection.

* Challenged groups, in plaque forming units (pfu). Diluent = PBS + .05%BSA. GAMIR = gamma irradiated Variola virus.

˟ pi = days post inoculation, day of sample collection.

ˠ Sera were tested at 1:50, 1:200, 1:800 and 1:3200 dilutions.

**Table 4:** Comparison of clinical signs presentation on CAST/EiJ mice infected with different doses of *Variola virus* during Phase 2 (animals challenged at 5–6 weeks of age).

Dosage	5 × 10^3^*	5 × 10^4^*	5 × 10^5^*	5 × 10^6^*
Reduce grooming/pruritus onset (number of animals)	Day 15 pi (3/5)	Day 7 pi (3/5)	Day 7 pi (5/5)	Day 7 pi (5/5)
Oral/nasal erythema (number of animals)	NO	Day 7 pi (3/5)	Day 7 pi (2/5)	Day 7 pi (3/5)
Respiratory distress under anesthesia (number of animals)	NO	NO	Day 9 pi (4/5)	Day 9 pi (3/5)
Ocular discharge (number of animals)	Day 9 pi (1/5)	Day 7 pi (2/5)	Day 7 pi (3/5)	Day 7 pi (3/5)
Rear paw/tail skin lesions (number of animals)	NO	NO	Day 8 pi (2/5)	Day 9 pi (2/5)NP
Completed recovery	Day 17 pi	Day 19 pi	Day 19 pi	Day 19 pi

Mice from gamma irradiated VARV and vehicle (PBS + .05% BSA) groups did not show any clinical signs mentioned above.

pi = days post inoculation.

NO = Never observed.

NP = Negative by real time PCR.

* Challenged groups, in plaque forming units (pfu).

**Table 5:** CAST/EiJ mice positive samples by real time PCR and Tissue Culture from the Phase 2 of the study (animals challenged *Variola virus* at 5–6 weeks of age).

ID#	Group*	Day pi^˟^	Sample	PCR^ˠ^	pfu/mL
20	5×l0^3^	9	oral swab	38.4/38.1	70
6	5×l0^4^	11	oral swab	38.3/38.3	BLD
7	5×10^4^	9	oral swab	37.3/38.5	BLD
8	5×10^4^	9	oral swab	38.1/38.4	BLD
8	5×l0^4^	11	oral swab	39.1/38.2	BLD
10	5×10^4^	9	oral swab	38.1/37.3	BLD
11	5×10^5^	7	oral swab	39.9/39.9	BLD
11	5×10^5^	9	oral swab	35.1/34.5	55
11	5×10^5^	11	tail lesion swab	36.9/37.3	BLD
11	5×10^5^	11	oral swab	37.6/37.6	BLD
11	5×10^5^	14	oral swab	39.3/39.3	BLD
12	5×10^5^	7	oral swab	36.1/35.9	417.5
12	5×10^5^	9	oral swab	35.4/36.4	52.5
12	5×10^5^	11	oral swab	38.7/38.1	BLD
13	5×10^5^	4	oral swab	39.9/39.9	65
13	5×10^5^	7	oral swab	34.7/34.8	550
13	5×10^5^	15	lung	37.1/37.0	BLD
14	5×10^5^	7	oral swab	39.1/37.9	140
14	5×10^5^	8	tail lesion swab	37.9/38.1	BLD
14	5×10^5^	9	oral swab	37.8/37.5	BLD
15	5×10^5^	7	oral swab	37.3/37.8	BLD
1	5×l0^6^	7	oral swab	35.1/35.2	395
1	5×l0^6^	9	oral swab	32.4/31.9	75
1	5×l0^6^	11	oral swab	38.7/38.7	BLD
2	5×l0^6^	7	oral swab	38.5/38.2	BLD
2	5×l0^6^	9	oral swab	35.0/35.8	102.5
3	5×l0^6^	7	oral swab	35.9/35.8	500
3	5×l0^6^	11	oral swab	38.6/37.8	BLD
4	5×l0^6^	7	oral swab	36.7/36.6	432.5
4	5×l0^6^	9	oral swab	33.6/33.7	287.5
4	5×l0^6^	11	oral swab	35.0/35.7	BLD
5	5×l0^6^	4	oral swab	39.6/39.0	BLD
5	5×l0^6^	7	oral swab	36.8/36.5	BLD
5	5×l0^6^	9	oral swab	36.9/36.1	BLD

* Challenged groups, in plaque forming units (pfu).

˟ pi = days post inoculation, day of sample collection.

ˠ Duplicates of the positive values, number of cycles needed to cross the threshold (CT).

" Viable virus content per mL, in pfu. BLD = below limit of detection.

Samples containing viable virus are shaded in grey.

Samples from gamma irradiated VARV and vehicle (PBS + .05%BSA) groups, did not show evidence of detectable virus either by real time PCR or Tissue Culture.

**Table 6:** Immune response of 5–6 weeks old CAST/EiJ mice from Phase 2, after challenge with different doses of *Variola virus*.

ID #	Group*	Age	Serum^˟^	Positive Dilution^ˠ^
24	Diluent	5 weeks old	21 days pi	BLD
25	Diluent	5 weeks old	21 days pi	BLD
26	Diluent	5 weeks old	21 days pi	BLD
21	GAMIR	5 weeks old	21 days pi	BLD
22	GAMIR	6 weeks old	21 days pi	BLD
23	GAMIR	5 weeks old	21 days pi	1:50
16	5 × 10^3^	6 weeks old	21 days pi	> 1:3200
17	5 × 10^3^	6 weeks old	21 days pi	> 1:3200
18	5 × 10^3^	5 weeks old	21 days pi	> 1:3200
19	5 × 10^3^	5 weeks old	21 days pi	NT^"^
20	5 × 10^3^	6 weeks old	21 days pi	> 1:3200
6	5 × 10^4^	6 weeks old	21 days pi	> 1:3200
7	5 × 10^4^	6 weeks old	21 days pi	> 1:3200
8	5 × 10^4^	6 weeks old	21 days pi	> 1:3200
9	5 × 10^4^	5 weeks old	21 days pi	> 1:3200
10	5 × 10^4^	5 weeks old	21 days pi	> 1:3200
11	5 × 10^5^	5 weeks old	21 days pi	> 1:3200
12	5 × 10^5^	6 weeks old	21 days pi	> 1:3200
13	5 × 10^5^	6 weeks old	16 days pi	> 1:3200
14	5 × 10^5^	5 weeks old	21 days pi	> 1:3200
15	5 × 10^5^	6 weeks old	21 days pi	> 1:3200
1	5 × 10^6^	6 weeks old	21 days pi	> 1:3200
2	5 × 10^6^	6 weeks old	21 days pi	> 1:3200
3	5 × 10^6^	6 weeks old	21 days pi	> 1:3200
4	5 × 10^6^	5 weeks old	21 days pi	> 1:3200
5	5 × 10^6^	5 weeks old	21 days pi	> 1:3200

* Challenged groups, in plaque forming units (pfu). Diluent = PBS + .05%BSA. GAMIR = gamma irradiated Variola virus.

˟ pi = days post inoculation, day of sample collection.

ˠ Sera were tested at 1:50, 1:200, 1:800 and 1:3200 dilutions. BLD = below limit of detection.

" NT = No sample available to test, animal was found dead on day 15 pi.
